# Trauma recidivism: epidemiology and predictors in a level I trauma center

**DOI:** 10.1186/s40621-026-00683-4

**Published:** 2026-05-21

**Authors:** Maheen Ibrahimi, Ahmed Hamed, Callie Hlavin, Tamara Byrd, Emilia J. Diego, Joshua Brown, Jason Sperry, Mazen S. Zenati

**Affiliations:** 1 University of Pittsburgh School of Medicine, Pittsburgh, PA USA; 2 Department of Surgery, Department of Surgery, University of Pittsburgh School of Medicine, University of Pittsburgh School of Medicine, 200 Lothrop Street, Pittsburgh, PA 15213 USA

**Keywords:** Trauma, Recidivism, Geriatric, Fall prevention

## Abstract

**Background:**

Trauma is the leading cause of death for individuals under age 45 and disproportionately affects underserved populations. Trauma recidivism is defined as recurrent hospital admissions for injury—a substantial burden on healthcare systems. In regions such as Allegheny County, Pennsylvania, which has a large, aging population, recidivism is often influenced by social, behavioral, and structural determinants. This study examines patterns of trauma recidivism at an urban tertiary trauma center and identifies associated risk factors to guide targeted interventions in both hospital and community settings.

**Methods:**

We conducted a retrospective cohort study using a trauma registry at a level I urban trauma center, including 13,967 patients admitted between January 2021 and September 2024. Patients were categorized as recidivists (*n* = 245) or non-recidivists (*n* = 13,455) based on repeated trauma admissions due to the same mechanism of injury. Demographic, injury, hospital course, and discharge variables were compared between recidivists and non-recidivists using chi-square tests, t-tests, or nonparametric equivalents as appropriate. Univariate and multivariate logistic regression analyses were performed to identify independent predictors of recidivism.

**Results:**

Recidivists accounted for 3.7% of the cohort. Compared to non-recidivists, recidivists were older (mean age 68.5 vs. 55.7 years, *p* < 0.001) and more likely to be female (53.9% vs. 40.5%, *p* < 0.001). Falls were the predominant injury mechanism (84.6% vs. 52.6%, *p* < 0.001), frequently occurring in home (56.4%, *p* < 0.001), particularly in bedrooms and bathrooms. Recidivists were less often discharged home (42.8% vs. 63.2%, *p* < 0.001). Risk of recidivism was higher among patients with injuries due to falls and self-harm, older age, female sex, and those discharged against medical advice or to a drug and alcohol rehabilitation facility.

**Conclusions:**

Trauma recidivism is increasingly driven by older adults with blunt injuries, highlighting a shift in the at-risk population. Prevention efforts should adapt to focus on age-related risks such as falls and frailty. Tailored discharge planning, psychosocial support, and post-discharge follow-up care may help reduce recurrent injury and improve outcomes in this growing patient group.

**Level of Evidence:**

Level IV, Prognostic/Epidemiological.

**Supplementary Information:**

The online version contains supplementary material available at 10.1186/s40621-026-00683-4.

## Introduction

Trauma is the leading cause of death among individuals under the age of 45 in the United States, contributing significantly to years of potential life lost and long-term disability [1]. Beyond its immediate mortality impact, trauma imposes a profound economic burden, with annual injury-related costs exceeding $400 billion in healthcare expenditures [2].

A subset of these cases involves trauma recidivism–repeated injuries requiring hospital-based care–which represents a significant yet underrecognized public health concern. Recidivism not only endangers individual health and safety but also strains already overburdened trauma systems [3]. In urban trauma care center settings, it is estimated that up to 20% of trauma patients have a subsequent injury requiring hospitalization within a year of the initial injury, and the risk of death increases nearly two-fold with each additional injury-related hospital visit [4–6].

Trauma recidivism can be associated with sociodemographic disparities. Many studies have shown that underserved populations, including racial and ethnic minorities and individuals of low socioeconomic status, are at a heightened risk of recurrent injury [7]. Structural determinants such as housing instability, unemployment, and limited access to healthcare further perpetuates this cycle [8]. In urban areas, these dynamics are exacerbated by community-level factors, including substance use and incarceration, resulting in higher recidivism rates [9].

Behavioral and mental health conditions are also powerful contributors. Patients who have reported alcohol or illegal drug use on the day of injury or who are victims or perpetrators of interpersonal violence have higher rates of recidivism compared to other mechanisms of trauma [10, 11]. Mental illness in particular has emerged as a key predictor: in a 2023 study evaluating trauma recidivism after orthopedic injury, mental illness represented the greatest risk factor for recurrent trauma admissions, seen in 57% of recurrent trauma admissions [12]. Similarly, among young patients, firearm violence survivors are at increased risk of repeat injury, which reflects continued exposure to high-risk environments [13].

Historically, trauma recidivism has been extensively studied in younger populations, often due to the associations with interpersonal violence, child abuse, and socioeconomic disadvantages [14]. However, emerging evidence now suggests that older adults are also at significant risk, primarily due to falls, which are the leading cause of injury-related hospitalization in this population and have been linked to worse long-term survival in those with recurrent falls compared to those who fall only once [15, 16]. Falls in the elderly are often multifactorial, resulting from physiological factors (e.g., balance impairment, chronic illnesses) compounded by environmental hazards and ongoing behavioral health needs, such as depression and dementia [17]. In regions such as Allegheny County, Pennsylvania, where the population is aging rapidly, trauma recidivism among older adults presents as a distinct challenge.

Integrated interventions such as fall prevention programs, home safety assessments, and constructed post-discharge follow-up can reduce recurrence and improve outcomes [18]. For instance, a stepped-care mental health program at a level I trauma center significantly reduced recidivism rates by focusing on psychosocial interventions [19]. Similarly, trauma survivor networks providing coping skills and self-efficacy have demonstrated reduction in repeat injury and substantial cost savings for healthcare systems [20].

Nevertheless, there is a need for approaches that extend beyond the hospital and address both psychosocial aspects and community-level determinants of recurrent trauma.

The present study aims to address this gap by analyzing trauma recidivism among patients admitted to an urban tertiary trauma center within the University of Pittsburgh Medical Center (UPMC). We hypothesize that trauma recidivism will be associated with distinct patient and injury characteristics, allowing for identification of high-risk subgroups for repeat injury, where we seek to identify modifiable risk factors and inform effective hospital and community-based intervention strategies.

## Methods

### Study design

We conducted a retrospective cohort study to evaluate trauma recidivism among patients admitted at an urban level I trauma center within the UPMC health system, located in Allegheny County, Pennsylvania. The trauma center serves a diverse population across the tri-state area (Pennsylvania, Ohio, and West Virginia) and maintains a comprehensive trauma registry compliant with state and national reporting requirements, which was used as the primary data source. The study was reported in accordance with STROBE guideline for observational research (Supplementary Digital Content 1).

### Setting

The study was conducted at a single urban tertiary care trauma center with a broad referral base encompassing both urban and rural communities. The study period spanned from January 1, 2021, to September 30, 2024. This interval covered recruitment (trauma admission), exposure (initial trauma and recurrent trauma admissions), follow-up (longitudinal tracking of subsequent admissions), and data collection (trauma registry), and was selected based on available registry data.

### Participants

Using the trauma registry, we identified 13,967 unique trauma admissions. Inclusion criteria were hospital admission for traumatic injury, length of hospital stay greater than 24 h, and injury severity score (ISS) greater than 8. Exclusion criteria were non-traumatic causes and patients with an ISS less than 8, and those who died at first injury. Patients were categorized into two groups:

- Recidivists: individuals with repeated trauma admissions due to the same mechanism of injury within the study period.
- Non-recidivists: Individuals with a single qualifying trauma admission.

Patients who were admitted more than once with different mechanisms of injury did not qualify for the recidivism definition. Follow-up for recidivism was conducted within the registry across the study period, and only those who met inclusion criteria were included in the dataset.

### Variables

The primary outcome was trauma recidivism (binary: recidivist vs. non-recidivist). Predictor variables included age, sex, race, etiology of injury, ISS, length of stay, and discharge disposition. Potential confounders include socioeconomic status, comorbidities, substance use, and insurance status. Effect modifiers may include age group and mechanism of injury. Diagnostic criteria for trauma followed the Pennsylvania Trauma Systems Foundation (PTSF).

### Data sources and measurement

All data were extracted from the UPMC trauma registry, which included standardized, validated fields across demographic, clinical, and outcome domains. Measurement methods were uniform across all variables and adhered to PTSF specifications.

### Bias

Data entry followed consistent registry protocols, thereby minimizing information bias and optimizing comparability between groups. All eligible admissions meeting the inclusion criteria during the study period were analyzed. Misclassification bias was mitigated by enforcing consistent injury etiology for recidivism classification. However, residual confounders due to unmeasured variables, such as socioeconomic status or comorbidity burden remain possible and acknowledged as a limitation.

### Statistical methods

Comparative analyses between recidivists and non-recidivists were conducted using descriptive statistics. Continuous variables were summarized as means with standard deviations (SD) or median with interquartile ranges (IQR) and compared using Student’s t tests or the Wilcoxon Rank-Sum (Mann-Whitney U test) as appropriate. Categorical variables were summarized as frequencies and percentages and compared using χ² or Fisher’s exact tests. Paired statistical methods were used where appropriate, including paired t-tests and Wilcoxon signed-rank tests. A statistical significance level of *P* < 0.05 was used for all comparisons. Univariate and multivariate logistic regression models were constructed to identify independent predictors of trauma recidivism. Variables with *p* < 0.15 in univariate analysis or deemed clinically relevant were entered into the multivariate model. Missing data were minimal, and in cases where fields were missing, complete case analysis was performed. Loss to follow-up was addressed by solely relying on the trauma registry which captures all subsequent admissions within the study period; however, patients presenting to other facilities may not be captured. All potential recidivism events underwent manual verification using multiple patient identifiers and review of injury summaries to ensure accurate case classification.

## Results

### Study population

After applying inclusion criteria (trauma-related admission, ISS > 8, hospital stay > 24 h), all 13,967 trauma admissions were included in the final cohort. Of these, 13,455 (96.3%) represented single (non-recurrent) admission, and 512 (3.7%) met the criteria for trauma recidivism with at least one episode of trauma recidivism. For analyses between recidivism and non-recidivism admissions, we restricted our subgroup to recidivists at their first admission (*n* = 245), compared to the 13,455 admissions of non-recidivists. There were no instances of non-participation since there was no active consent process, as this was a retrospective registry-based study.

### Descriptive data

Table 1 provides demographic and geographic characteristics for the entire cohort (*n* = 13,967), non-recidivists (*n* = 13,455), and those recidivists at their first admission (*n* = 245). Recidivists were significantly older (mean age at injury 67.8 ± 18.8 vs. 55.2 years ± 21.4; *p* < 0.001), and more likely to be female (*n* = 133, 54.3% vs. *n* = 5454, 40.5%; *p* < 0.001). Racial distribution differed modestly, with a higher proportion of White patients among the recidivists (*n* = 215, 87.8% vs. *n* = 10919, 81.2%; *p* = 0.010). Patients from Allegheny County (*n* = 167, 68.2%; *p* < 0.001) and the greater Pittsburgh area (*n* = 226, 92.2%; *p* < 0.001) were also disproportionately represented among recidivists. No missing data was reported. Given the retrospective nature of the study, follow-up was determined by the study window (Jan 2021 to Sept 2024), during which all patients were tracked for repeat admissions. Table 2 provides characteristics on patient injury between recidivists and non-recidivists, and Table 3 provides characteristics of outcomes between recidivists and non-recidivists. Table 4 provides characteristics on patient injury and outcomes based on admission episode. Table 5 provides injury etiology stratified by age and recidivism status.

**Table 1:** Demographic Characteristics of Cohort based on Recidivism

Characteristic	Cohort (*n* = 13967)	No Recidivism (*n* = 13455, 96.3%)	Recidivism (*n* = 245, 1.8%)	*P*-value
Patient Demographics				
Age at injury	55.7 ± 21.4	55.2 ± 21.4	67.8 ± 18.8	< 0.001
Age at ED admission	56.2 ± 21.4	55.7 ± 21.4	68.3 ± 18.8	< 0.001
Gender				
Male	8236 (59.0)	8002 (59.5)	112 (45.7)	< 0.001
Female	5731 (41.0)	5454 (40.5)	133 (54.3)	
Race				
White	11,372 (81.4)	10,919 (81.2)	215 (87.8)	0.010
Black	1936 (13.9)	1887 (14.0)	24 (9.8)	
Asian & Others	265 (1.9)	261 (1.9)	2 (0.8)	
Unknown	394 (2.8)	389 (2.9)	4 (1.6)	
Alleghany County	6500 (46.5)	6145 (45.7)	167 (68.2)	< 0.001
State				
OH	750 (5.4)	745 (5.5)	2 (0.8)	< 0.001
PA	12,505 (89.5)	12,005 (89.2)	240 (98.0)	
WV	378 (2.7)	372 (2.8)	3 (1.2)	
Unknown	12 (0.1)	12 (0.1)	0	
Out of tri state area	322 (2.3)	322 (2.4)	0	
Greater Pittsburgh & Surrounding Counties	10,760 (77.0)	10,288 (76.5)	226 (92.2)	< 0.001

Demographic characteristics are shown for the overall cohort and stratified by patients without recidivism and those with trauma recidivism. Continuous variables are presented as mean ± standard deviation, and categorical variables are presented as number (percentage). P-values reflect comparisons between recidivists and non-recidivists using *t* test for continuous variables and χ² tests for categorical variables. Statistically significant differences were observed across multiple demographics, including age, sex, race, and geographic residence

**Table 2:** Injury Characteristics based on Recidivism

Characteristic	Cohort (*n* = 13967)	No Recidivism (*n* = 13455, 96.3%)	Recidivism (*n* = 245, 1.8%)	*P*-value
Injury				
ISS	9 (4–13)	9 (4–13)	9 (4–12)	0.946
Etiology				
Fall	7512 (53.8)	7078 (52.6)	209 (85.3)	< 0.001
Self-Harm	322 (2.3)	293 (2.2)	13 (5.3)	
MVA	3236 (23.2)	3211 (23.9)	12 (4.9)	
Homicide/Assault	1043 (7.5)	1024 (7.6)	9 (3.7)	
Natural	205 (1.5)	205 (1.5)	0	
Overexertion	43 (0.3)	43 (0.3)	0	
Undetermined Accident	62 (0.4)	62 (0.5)	0	
Accident with Objects	1029 (7.4)	1025 (7.6)	2 (0.8)	
Other	200 (1.4)	200 (1.5)	0	
Unknown	315 (2.3)	315 (2.3)	0	
Place of Injury				
Home	5037 (36.1)	4747 (35.3)	151 (61.6)	< 0.001
Road	3112 (22.3)	3068 (22.8)	21 (8.6)	
Steps	413 (3.0)	397 (3.0)	9 (3.7)	
Healthcare Facility	444 (3.2)	391 (2.9)	20 (8.2)	
Workplace	80 (0.6)	78 (0.6)	0	
Restaurant	177 (1.3)	176 (1.3)	0	
Shops	203 (1.5)	196 (1.5)	3 (1.2)	
Recreation	362 (2.6)	360 (2.7)	2 (0.8)	
Outdoors	378 (2.7)	372 (2.8)	4 (1.6)	
Parking Lots	183 (1.3)	179 (1.3)	3 (1.2)	
Industrial	129 (0.9)	129 (1.0)	0	
School/Worship	62 (0.4)	60 (0.5)	1 (0.4)	
Prison	64 (0.5)	61 (0.5)	1 (0.4)	
Unspecified/Other	3323 (23.8)	3242 (24.1)	30 (12.2)	
Home injury location				
Bedroom	535 (10.6)	488 (10.3)	17 (11.3)	< 0.001
Bathroom	625 (12.4)	579 (12.2)	26 (17.2)	
Kitchen/Dining Room	393 (7.8)	357 (7.5)	19 (12.6)	
Living area	277 (5.5)	256 (5.4)	13 (8.6)	
Outdoors	733 (14.6)	712 (15.0)	11 (7.3)	
Garage	291 (5.8)	279 (5.9)	7 (4.6)	
Steps	270 (5.4)	256 (5.4)	10 (6.6)	
Basement/Attic	101 (2.0)	96 (2.0)	2 (1.3)	
Unspecified/General home	1811 (36.0)	1723 (36.3)	46 (30.5)	
Injury type				
Blunt	12,310 (88.2)	11,837 (88.0)	227 (92.7)	0.251
Penetrating	1621 (11.6)	1580 (11.8)	18 (7.4)	
Burn	28 (0.2)	28 (0.2)	0	
Skin disease	1 (0.0)	1 (0.0)	0	
Tourniquet use	361 (2.7)	354 (2.8)	3 (1.3)	0.460
Extrication	1347 (10.8)	1305 (10.9)	18 (7.9)	0.533
Systolic blood pressure	138.8 ± 26.8	138.6 ± 26.7	144.1 ± 27.2	0.022
Respiratory rate	18 (16–20)	18 (16–20)	18 (16–20)	0.543
Vent Days (for those vented)	2 (1–7)	2 (1–7)	2 (1–14)	0.530
ED CPR	44 (1.9)	43 (2.0)	1 (2.7)	0.634
Base Deficit	4 (2–8)	4 (2–8)	4 (1–8)	0.920
Whole Blood transfusion	554 (4.0)	547 (4.1)	1 (0.4)	0.004
Amount (mL)	1000 (500–1100)	1000 (500–1100)	500 (500–500)	0.325
PRBCs transfused (mL)	501 (3.6)	489 (3.6)	5 (2.0)	0.552
Amount (mL)	600 (500–1300)	600 (500–1300)	600 (600–1200)	0.861
Total Prehospital Fluids Administered				
None	6536 (53.4)	6248 (52.9)	125 (60.7)	< 0.001
<500 mL	1331 (10.9)	1303 (11.0)	16 (7.8)	
500–2000 mL	1245 (10.2)	1227 (10.4)	7 (3.4)	
>2000 mL	212 (1.7)	207 (1.8)	1 (0.5)	
Infused, amount unknown	2925 (23.9)	2826 (23.9)	57 (27.7)	
SaO2 on admission	98 (96–99)	98 (96–99)	97 (95–99)	0.005
Pulse on admission	89.5 ± 19.1	86.5 ± 19.2	85.7 ± 17.8	0.399
Temperature (c)	36.8 ± 0.6	36.8 ± 0.6	36.8 ± 0.4	0.078

Injury characteristics are presented for the overall cohort and stratified by patients without recidivism and those with trauma recidivism. Continuous variables are reported as mean ± standard deviation or median with IQR, and categorical variables as number (percentage). Comparisons between recidivists and non-recidivists were performed using *t* test or Wilcoxon rank-sum test for continuous variables and χ² or Fisher’s exact tests for categorical variables. P-values reflect between-group comparisons

**Table 3:** Outcome Characteristics based on Recidivism

Characteristic	Cohort (*n* = 13967)	No Recidivism (*n* = 13455, 96.3%)	Recidivism (*n* = 245, 1.8%)	*P*-value
ICU length of stay (days)	2 (1–5)	2 (1–5)	2 (1–3)	0.036
Time in ED (minutes)	308 (195–498)	307 (194–497)	352 (228-500.5)	< 0.001
Hospital length of stay (days)	4 (1–8)	4 (1–8)	5 (2–8)	< 0.001
Discharge Destination				
AMA	187 (1.8)	178 (1.3)	5 (2.0)	< 0.001
Healthcare Facility	3458 (24.8)	3227 (24.0)	104 (42.5)	
Rehab	890 (6.4)	851 (4.3)	18 (7.4)	
Home/Homeless	8742 (62.6)	8525 (63.4)	116 (47.4)	
Legal Authority	128 (0.9)	124 (0.9)	2 (0.8)	
Unspecified	562 (4.0)	551 (4.1)	0	
Discharged home from ED	8723 (62.5)	8503 (63.2)	116 (47.4)	< 0.001
Vital status				
Alive	13,406 (96.0)	12,903 (95.9)	245 (100.0)	0.015
Dead	561 (4.0)	550 (4.1)	0	
Any Surgery	1073 (7.7)	1047 (7.8)	10 (4.1)	0.124

Hospital utilization, discharges, and clinical outcomes are shown for the overall cohort and stratified by recidivism status. Continuous variables are presented as median with IQR, and categorical variables as number (percentage). Statistical comparisons between recidivists and non-recidivists were conducted using Wilcoxon rank-sum tests for continuous variables and χ² or Fisher’s exact tests for categorical variables. ED delineates emergency department; ICU, intensive care unit; AMA, against medical advice

**Table 4:** Injury and Outcome Characteristics based on Number of Episodes of Recidivism

Characteristic	Cohort (*n* = 514)	First Episode (*n* = 245, 47.7%)	Second Episode (*n* = 245, 47.7%)	Additional episodes (*n* = 24, 4.7%)	*p*-value
Injury					
ISS	9 (4–12)	9 (4–12)	9 (4–12)	9 (2–11)	0.946
Etiology					
Fall	435 (84.6)	209 (85.3)	208 (84.9)	17 (81.0)	< 0.001
Self-Harm	29 (5.6)	13 (5.3)	13 (5.3)	3 (14.3)	
MVA	27 (5.3)	12 (4.9)	13 (5.3)	0	
Homicide/Assault	19 (3.7)	9 (3.7)	9 (3.7)	1 (4.8)	
Accident with Objects	4 (0.8)	2 (0.8)	2 (0.8)	0	
Injury at home	5036 (36.1)	151 (61.6)	127 (51.8)	12 (57.1)	< 0.001
Injury type					
Blunt	473 (92.0)	227 (92.7)	223 (91.0)	20 (95.2)	0.495
Penetrating	41 (8.0)	18 (7.4)	22 (9.0)	1 (4.8)	
Tourniquet use	7 (1.5)	3 (1.3)	4 (1.8)	0	0.339
Extrication	42 (8.8)	18 (7.9)	22 (9.7)	2 (9.5)	0.497
GCS	15 (14–15)	15 (14–15)	15 (14–15)	14.5 (14–15)	0.075
Mean/SD	14.4 ± 1.9	14.5 ± 1.6	14.2 ± 2.2	14.6 ± 0.5	0.064
Systolic blood pressure	143.0 ± 28.3	144.1 ± 27.2	142.0 ± 29.4	143.2 ± 31.5	0.022
Respiratory rate	18 (16–20)	18 (16–20)	18 (16–20)	17 (15–19)	0.543
Vent Days	2 (1-4.5)	2 (1–14)	2 (1–3)	11 (11–11)	0.530
ED CPR	1 (1.2)	1 (2.7)	0	0	0.634
Transferred from care facility	216 (42.0)	114 (46.5)	94 (38.4)	5 (23.8)	0.634
Base Deficit	4 (1–8)	4 (1–8)	4 (1-15.1)	-	0.920
Whole Blood transfused (mL)	7 (1.4)	1 (0.4)	6 (2.5)	0	0.004
Amount (mL)	500 (500–1100)	500 (500–500)	750 (500–1000)	-	0.325
PRBCs transfused (mL) (yes)	12 (2.3)	5 (2.0)	7 (2.9)	0	0.552
Amount (mL)	600 (325–1100)	600 (600–1200)	600 (325–1000)	-	0.861
Total Prehospital Fluids Administered					
None	288 (65.8)	125 (60.7)	147 (70.0)	16 (84.2)	< 0.001
<500 mL	28 (6.4)	16 (7.8)	11 (5.2)	1 (5.3)	
500–2000 mL	18 (4.1)	7 (3.4)	10 (4.8)	1 (5.3)	
>2000 mL	5 (1.1)	1 (0.5)	4 (1.9)	0	
Infused, amount unknown	99 (22.6)	57 (27.7)	38 (18.1)	1 (5.3)	
SaO2 on admission	97 (95–99)	97 (95–99)	97 (95–99)	97 (94.5–98.5)	0.005
Pulse on admission	85.4 ± 17.6	85.7 ± 17.8	85.3 ± 17.3	84.3 ± 19.0	0.399
Temperature ©	36.7 ± 0.4	36.8 ± 0.4	36.7 ± 0.4	36.8 ± 0.5	0.078
Outcomes					
ICU length of stay (days)	2 (1–3)	2 (1–3)	2 (1–3)	12 (12–12)	0.036
Time in ED	357 (223–544)	352 (228-500.5)	358 (222–563)	421 (213–751)	< 0.001
Hospital length of stay (days)	5 (2–8)	5 (2–8)	5 (3–9)	5 (2–6)	< 0.001
Discharge destination					
In-Hospital	472 (91.8)	221 (90.2)	229 (93.5)	19 (90.5)	0.015
Transfer	20 (3.9)	9 (3.7)	9 (3.7)	2 (9.5)	
Home	19 (3.7)	13 (5.3)	6 (2.5)	0	
Other	2 (0.4)	2 (0.8)	0	0	
Prison	1 (0.2)	0	1 (0.4)	0	
Vital status					
Alive	503 (97.9)	245 (100.0)	235 (95.9)	20 (95.2)	0.015
Dead	11 (2.1)	0	10 (4.1)	1 (4.8)	
Any Surgery	26 (5.1)	10 (4.1)	15 (6.1)	1 (4.8)	0.124
Discharge destination					
AMA	9 (1.8)	5 (2.0)	3 (1.2)	1 (4.8)	< 0.001
Healthcare Facility	231 (45.2)	104 (42.5)	116 (47.4)	11 (52.4)	
Rehab	39 (7.6)	18 (7.4)	19 (7.8)	2 (9.5)	
Home/Homeless	217 (42.5)	116 (47.4)	95 (38.8)	6 (28.6)	
Legal Authority	4 (0.8)	2 (0.8)	2 (0.8)	0	
Unspecified	11 (2.2)	0	10 (4.1)	1 (4.8)	

Injury patterns and hospital outcomes are shown for all recidivism-related admissions and stratified by first, second, and subsequent episodes of trauma recidivism. Continuous variables are reported as mean ± standard deviation or median with IQR, and categorical variables as number (percentage). P-values reflect comparisons across all recidivism episodes

**Table 5:** Injury Etiology Stratified by Age and Recidivism Status

Characteristic	Cohort (*n* = 13967)	No Recidivism (*n* = 13455, 96.3%)		Recidivism (*n* = 245, 1.8%)		*p*-value
		Age < 65	Age$\:\ge\:$65	Age < 65	Age$\:\ge\:$65	
Etiology						
Fall	7512 (53.8)	2929 (35.0)	4148 (81.7)	102 (58.0)	333 (98.5)	< 0.001
Self-Harm	322 (2.3)	269 (3.2)	24 (0.5)	29 (16.5)	0	0.147
MVA	3236 (23.2)	2669 (31.9)	540 (10.6)	25 (14.2)	2 (0.6)	0.298
Homicide/Assault	1043 (7.5)	980 (11.7)	44 (0.9)	16 (9.1)	3 (0.9)	0.050
Natural	205 (1.5)	169 (2.0)	36 (0.7)	0	0	-
Overexertion	43 (0.3)	40 (0.5)	3 (0.1)	0	0	-
Undetermined Accident	62 (0.4)	59 (0.7)	3 (0.1)	0	0	-
Accident with Objects	1029 (7.4)	851 (10.2)	174 (3.4)	4 (2.3)	0	-
Other	200 (1.4)	132 (1.6)	68 (1.3)	0	0	-
Unknown	315 (2.3)	279 (3.3)	36 (0.7)	0	0	-

Distribution of injury etiologies for the cohort and stratified by recidivism status (no recidivism vs. recidivism) and age group (< 65 years and$\:\ge\:$65 years) is shown. Values are reported as counts with corresponding percentage. P-values were calculated using the Chi-square test of independence or Fisher’s exact test, as appropriate

### Injury characteristics

**Fig. 1:**
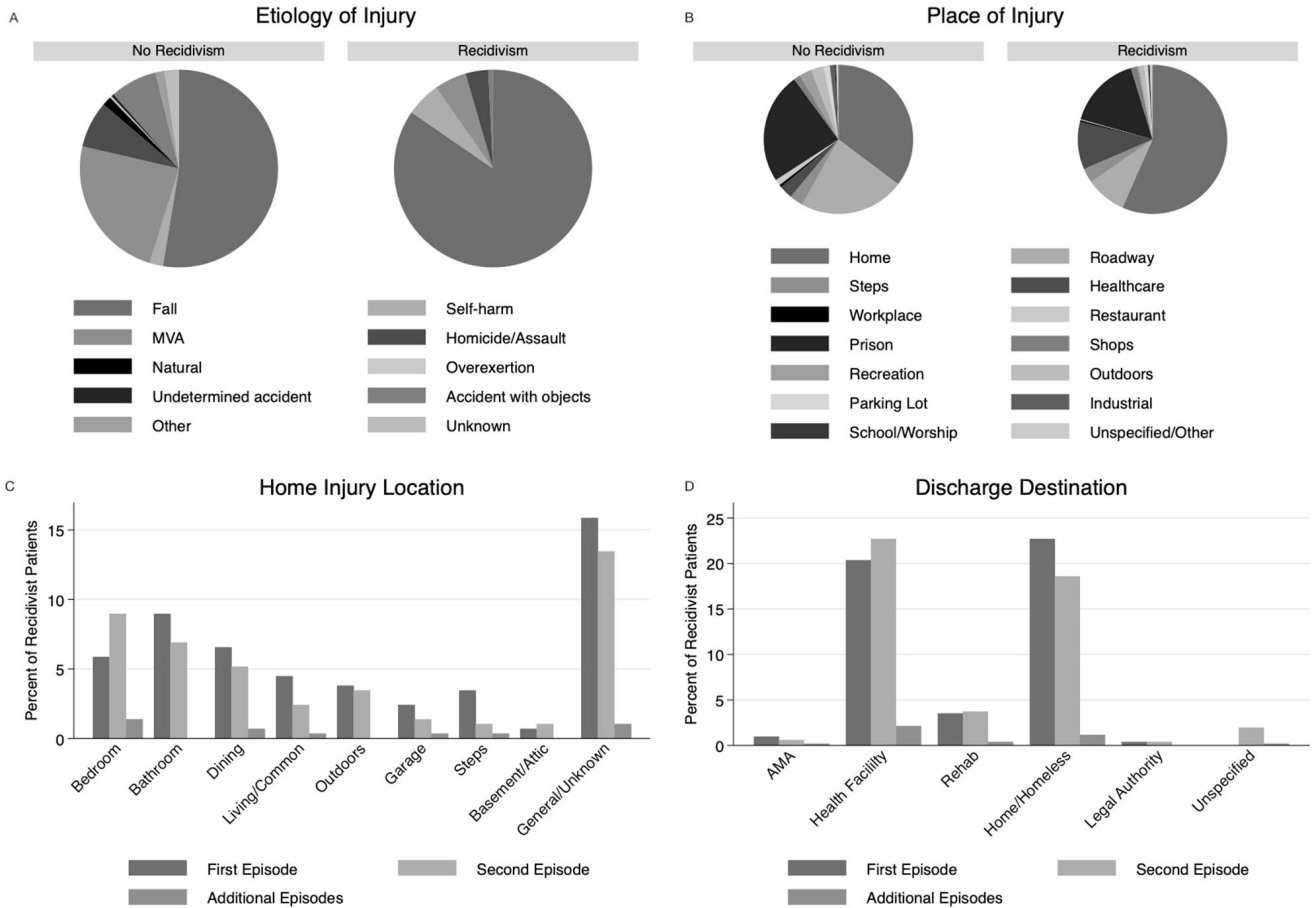
Injury etiology, location, and discharge patterns among patients with and without trauma recidivism. (**A**) Distribution of injury etiology among patients without recidivism and those with trauma recidivism at first admission. (**B**) Place of injury for non-recidivists and recidivists at first admission. (**C**) Home injury locations among recidivist patients, stratified by first, second, and additional recidivism episodes, displayed as a percentage of recidivist patients. (**D**) Discharge destinations following trauma-related admissions among recidivists, stratified by first, second, and additional episodes. Percentages are calculated within recidivist episodes. AMA indicates discharge against medical advice

Among the 245 recidivists included in the cohort, falls were the predominant mechanism of injury across all episodes of recidivism (*n* = 209, 85.3%) (Fig. 1A). In addition, those who were older (above age 65), had falls as mechanism of injury for repeated admissions compared to those less than age 65 (*n* = 333, 98.5% vs. *n* = 102, 58.0%). Self-harm accounted for 5.3% (*n* = 13) of first admission injuries, with an increased proportion (*n* = 3, 14.3%) in patients with more than two admissions; however, the sample size limits definitive interpretation. The ISS was similar across episodes of recidivism (median [IQR], 9 [4–12]; *p* = 0.946), with the majority of injuries being blunt (*n* = 227, 92.7%). Most recidivism injuries occurred at home (*n* = 151, 61.6%), with a higher proportion of home injuries during the first episode compared to the second (*n* = 151, 61.6% vs. *n* = 127, 51.8%, *p* < 0.001). Specifically, most of the recidivist injuries occurred in an unspecified or general home area (*n* = 46, 30.5%) (Fig. 1B), after which bedrooms and bathrooms were the most common location of injury (*n* = 17, 11.3% and *n* = 26, 17.2%, respectively; *p* < 0.001) (Fig. 1C).

### Hospital course

Recidivists had slightly longer emergency room stays (median [IQR], 352 [228-500.5] vs. 307 [194–497] minutes; *p* = 0.004) and hospital stays (median [IQR], 5 [2–8] vs. 4 [1–8] days; p = < 0.001), with additional episodes of recidivism showing even longer stays. ICU stays were similar with a median of 2 (IQR 1–3) across groups.

### Discharge outcomes

Recidivists were significantly less likely to be discharged home (*n* = 116, 47.4% vs. *n* = 8503, 63.2%; *p* < 0.001), decreasing with each subsequent episode (*n* = 116, 47.4% in the first, *n* = 95, 38.8% in the second, and *n* = 6, 28.6% in additional episodes; *p* < 0.001). They were also more likely to be discharged to a healthcare facility after each episode (*n* = 104, 42.5% in the first, *n* = 116, 47.4% in the second, *n* = 11, 52.4% in the additional episodes; *p* < 0.001). Mortality increased after the first episode—4.1% (*n* = 10) in the second and 4.8% (*n* = 1) in subsequent episodes (*p* = 0.015). This can be visualized in Fig. 1D.

### Univariate and multivariate analysis

To identify predictors of recidivism in trauma, we conducted univariate and multivariate logistic regression analyses (Table 6).

**Table 6:** Univariate and Multivariate Logistical Regression Analysis

	Univariate			Multivariate		
	OR	95% CI	*P* value	OR	95% CI	*P* value
**Injury Etiology**						
**All other causes**	1			1		
**Fall**	2.1	1.7–2.7	< 0.001	1.5	1.1–2.0	0.006
**Self-harm**	3.7	2.1–6.3	< 0.001	3.1	1.3–7.2	0.009
**Age in ED**	1.0	1.0–1.0	< 0.001	1.0	1.1–1022	< 0.001
**Sex (female)**	1.3	1.0–1.6	0.020	-		
**Race (Black)**	0.9	0.6–1.2	0.484	-		
**SBP**	1.0	1.0–1.0	0.014	-		
**GCS**	1.1	1.0–1.1	0.012	-		
**ISS**	1.0	1.0–1.0	0.197	-		
**Time in ED (minutes)**	1.0	1.0–1.0	< 0.001	1.0	1.0–1.0	0.041
**Transfer Patients**	1.2	1.0–1.5	0.072	1.3	1.0–1.7	0.050
**Discharge to** **All other destinations** **AMA** **Drug & Alcohol facility** **Psychiatric Facility** **Skilled nursing home**	1 2.8 9.6 2.6 2.0	1.5–5.4 1.1– 82.6 1.5–4.2 1.5–2.5	0.002 0.040 < 0.001 < 0.001	1 3.5 12.4 1.7 1.3	1.8–6.8 1.4–108 0.8–3.8 1.0–1.7	< 0.001 0.023 0.174 0.053

Univariate and multivariate logistic regression models were used to identify factors associated with trauma recidivism. Odds ratios (ORs) with 95% confidence intervals (CIs) are presented. ED delineates emergency department; SBP, systolic blood pressure; GCS, Glasgow Coma Scale; ISS, Injury Severity Score; AMA, against medical advice

In the univariate analysis, injury etiology emerged as a strong predictor. Patients injured by falls had over twice the odds of recidivism (OR 2.1, 95% CI 1.7–2.7; *p* < 0.001) and self-harm had almost four times the odds (OR 3.7, 95% CI 2.1–6.3; *p* < 0.001) compared to all other causes. These associations persisted in the multivariate model; self-harm and falls remained potent predictors of recidivism (falls: OR 1.50, 95% CI 1.1-2.0 *p* = 0.006, self-harm: OR 3.1, 95% CI 1.3–7.2; *p* = 0.009). Age at emergency department presentation was also positively associated with recidivism (multivariate OR 1.0, 95% CI 1.1–1022; *p* < 0.001). Being transferred from another facility also approached significance as a predictor (OR 1.3, 95% CI 1.0-1.7; *p* = 0.050). Other variables such as sex, race, systolic blood pressure, GCS, and ISS showed significance in the univariate models but were not retained in the final multivariate model.

Injury recidivists had higher odds of being discharged to non-home destination, specifically to drug and alcohol facilities. Compared to all other discharge destinations, patients being discharged to drug and alcohol rehabilitation (in multivariate analysis: OR 12.4, 95% CI 1.4–108; *p* = 0.023) and against medical advice (AMA) (OR 3.5, 95% CI 1.8–6.8; *p* < 0.001) had higher odds of recidivism. Discharge to psychiatric facilities was not a statistically significant risk factor despite an elevated odds ratio of 1.73 (95% CI 0.8–3.8; *p* = 0.174). Discharge to skilled nursing homes also was not statistically significant (OR 1.3, 95% CI 1.0-1.7; *p* = 0.053).

## Discussion

This study examined recidivism patterns within a large urban Level I trauma center and identified several independent predictors of recurrent injury. Recidivists were older and more likely to be female. Falls were the predominant cause of injury across all episodes of recidivism. Injuries also tended to occur at home, especially in high-risk environments such as bathrooms and bedrooms. Multivariate analysis identified fall and self-harm etiologies, increased time spent in the emergency department, and older age as independent predictors of recidivism. Discharge destinations that were drug and alcohol facilities and leaving AMA were also strongly correlated with repeat trauma injuries.

Our findings highlight the growing epidemiology of trauma recidivism, with older adults and females being the majority of recidivists—a shift from prior studies that primarily focus on young males and victims of interpersonal violence [11]. This discrepancy may depend on local population differences, as Allegheny County has a high proportion of elderly individuals, or may be due to evolution in trauma epidemiology. It also may suggest a broader spectrum of risk beyond violence exposure alone, considering that interpersonal violence, such as firearm injury, did not show a statistically significant association between injury mechanism and recidivism after adjustment. The predominance of falls as the injury mechanism among recidivists, however, reflects findings in earlier studies that note falls as the leading cause of injury-related hospitalizations in older adults [15, 21, 22]. This highlights the importance of tailored fall prevention strategies in geriatric populations and emphasizes the need for a primary care-based fall risk assessment and prevention, and early intervention services [17, 18, 23].

The association between recidivism and self-harm also deserves attention. Self-inflicted injuries accounted for a small proportion of recidivism events yet indicated that there was a nearly threefold increased risk for recidivism among those initially injured by self-harm. It is consistent with previous studies reporting that mental health is an underrecognized yet powerful contributor to recurrent trauma [12]. It underscores that trauma recidivism is as much a mental and social health issue as it is a physical one. For example, Rivas et al. [19] implemented a stepped-care mental health intervention, which significantly reduced trauma recurrence in high risk patients, highlighting the value of early behavioral health integration into trauma systems. Sinkler et al. [20] further emphasized the importance of psychosocial programming, suggesting that trauma care models may fail to address the root causes of repeated injury if they do not include mental health support. Our findings add to the call for routine behavioral health screening, trauma-informed care, and post-discharge psychosocial support for trauma patients.

Patients who were discharged to drug and alcohol facilities or who left against medical advice (AMA) had significantly higher odds of recidivism, echoing prior literature that shows that premature discharge and inadequate linkage to community support services perpetuate the cycle of injury [24, 25]. Glasgow et al. [25] suggest that hospitals should target AMA patients for follow-up, including phone calls and home visits, as well as offering counseling.

While this study includes a large, diverse urban trauma population over a three-year period, findings may not be fully generalizable to other regions, such as rural settings or non-level I trauma centers. UPMC has a wide catchment area, and patterns of recidivism may differ in communities with different socioeconomic, demographic, or healthcare infrastructure characteristics. However, with the inclusion of over 13,000 patients, it contributes to the literature and offers insights that may be relevant to similar tertiary trauma centers.

Strengths of this study include usage of a large, well-validated trauma registry from a high-volume level I trauma center, with consistent data definitions and inclusion criteria. The use of this registry minimizes misclassification and enhances internal validity. Our multivariate models also adjusted for relevant confounders and identified statistically significant predictors of recidivism. The time span of this study captures the longitudinal burden of recurrent trauma in a single health system. This allows for the identification of not only the initial recidivists but also those with three or more trauma visits, highlighting the persistence of recurrent injury patterns.

Compared to national estimates of trauma recidivism, which range from 7% to 20% [13, 26], our observed rate of 3.7% appears conservative. This discrepancy may reflect differences in follow-up duration, inclusion criteria, or potential under capture of patients who presented to other health care facilities such as a clinic or urgent care. It may also be driven by our narrow definition of recidivism, which may differ from previous studies: by capturing only repeat presentations for the same injury type, we may have undercounted patients who returned with different mechanisms or injury patterns. In addition, while the trauma registry provides rich clinical data, it lacks information on socioeconomic status, psychiatric diagnoses, and access to post-discharge services which may impact the recidivism risk. Lastly, the retrospective nature of our study precludes causal inference and may be subject to residual confounding.

Our findings underscore the need for injury prevention strategies in the older adult population. Evidence-based fall prevention programs, such as home hazard assessments, balance and gait training, and medication reconciliations, should be integrated into the trauma discharge process [27, 28]. Similarly, patients with self-harm injuries or discharge destinations may benefit with models such as the Trauma Recovery Services Program and stepped-care mental health interventions, which have shown success in reducing recidivism through psychosocial support and should be expanded [19, 29]. Health systems should also explore opportunities for risk stratification at the time of admission. For example, patients who arrive with fall-related injuries, previous self-harm, and older age may benefit from earlier involvement of social work, geriatrics, and psychiatry. Future work should focus on developing risk prediction models and evaluating the effectiveness of targeted interventions to reduce preventable injury and optimize recovery for trauma survivors.

## Conclusion

Trauma recidivism remains a pressing public health issue, yet a preventable problem within the trauma population. It is increasingly affecting older adults through mechanisms such as falls. Alcohol and substance abuse were among the strongest predictors of repeat injury. Our study provides demographic, clinical, and discharge-related factors associated with increased risk of recurrent injury in an urban academic medical center. Early identification of at-risk individuals and implementation of targeted prevention and rehabilitation programs at hospitals and community levels may reduce recurrence and improve long-term outcomes.

## Electronic Supplementary Material

Supplementary Material 1

## Data Availability

The datasets used and/or analysed during the current study are available from the corresponding author on reasonable request.

## Abbreviations

UPMC: University of Pittsburgh Medical Center
ISS: Injury severity score
PTSF: Pennsylvania Trauma Systems Foundation
AMA: Against medical advice
SD: Standard deviations
IQR: Interquartile ranges
SBP: Systolic blood pressure
GCS: Glasgow Coma Scale
CI: Confidence Interval
OR: Odds ratio
